# Association Between Lipid Profile and COVID-19 Severity: Insights from a Single-Center Cross-Sectional Study in Northern Greece

**DOI:** 10.3390/jcm14124082

**Published:** 2025-06-09

**Authors:** Athena Myrou, Konstantinos Barmpagiannos, Erofili Papathanasiou, Vasileios Kachtsidis, Christina Kiouli, Konstantinos Tziomalos

**Affiliations:** Department of Internal Medicine, AHEPA University General Hospital, Aristotle University of Thessaloniki, 54636 Thessaloniki, Greece; mparmpak@auth.gr (K.B.); eropapat@gmail.com (E.P.); billykachtsidis@gmail.com (V.K.); xristinaki516@gmail.com (C.K.); ktziomalos@yahoo.com (K.T.)

**Keywords:** COVID-19 severity, low-density lipoprotein, lipid metabolism, dyslipidemia, inflammation, C-reactive protein

## Abstract

**Objective:** To examine the relationship between lipid profile components—including low-density lipoprotein (LDL), high-density lipoprotein (HDL), and triglycerides—and clinical outcomes in hospitalized COVID-19 patients in Northern Greece. **Methods:** A retrospective analysis was performed using data from 208 COVID-19 patients. Lipid profiles [including LDL (low-density lipoprotein cholesterol), HDL (high-density lipoprotein cholesterol), and triglycerides], prior antilipidemic treatment, and clinical outcomes were evaluated. Statistical analysis was conducted using SPSS version 19. Patients: A total of 208 COVID-19 patients from Northern Greece. **Results:** The mean LDL level was 84.12 mg/dL, with no significant differences observed between survivors and non-survivors. Prior antilipidemic treatment did not significantly affect outcomes. Elevated triglyceride levels were noted in obese patients (BMI ≥ 30 kg/m^2^) and lower HDL levels were associated with higher CRP (C-reactive protein) levels. Although LDL levels declined over time in non-survivors, this decrease was not statistically significant. Longitudinal analysis showed normalization of LDL levels post-recovery, while HDL levels remained persistently low. **Conclusions:** Despite observable alterations in lipid profiles, their prognostic significance in this cohort was limited. These findings highlight the need for further investigation into the role of lipid metabolism in the pathophysiology of COVID-19.

## 1. Introduction

Since its emergence in late 2019, COVID-19 has posed unprecedented global health challenges, leading to millions of infections and fatalities worldwide. The disease, caused by severe acute respiratory syndrome coronavirus 2 (SARS-CoV-2), manifests across a broad spectrum of clinical presentations, from asymptomatic or mild symptoms to severe pneumonia, acute respiratory distress syndrome (ARDS), multi-organ failure, and death. The severity of COVID-19 is influenced by various host factors including age and comorbidities such as diabetes, cardiovascular diseases, obesity, and dysregulation of the immune response [1,2,3,4].

Emerging evidence suggests that lipid metabolism plays a pivotal role in the pathophysiology of viral infections, including SARS-CoV-2. Lipids are essential for viral entry, replication, and modulation of the immune function. Notably, low-density lipoprotein cholesterol (LDL-C) and high-density lipoprotein cholesterol (HDL-C) have been implicated in inflammatory regulation and endothelial function. Several studies have reported significant reductions in total cholesterol, LDL-C, and HDL-C levels during acute viral illnesses [5,6,7,8].

These disturbances may reflect systemic inflammation, hepatic dysfunction, altered lipoprotein metabolism, and direct viral interference with host lipid pathways [9,10,11,12]. In the context of COVID-19, a cytokine storm—characterized by elevated interleukin-6 (IL-6), tumor necrosis factor-alpha (TNF-α), and C-reactive protein (CRP)—has been proposed as a contributing factor to dyslipidemia. LDL-C levels tend to decline in severe cases, potentially due to increased catabolism, oxidative stress, or hepatic injury. Similarly, reduced HDL-C has been linked with impaired anti-inflammatory and antioxidant functions, which may exacerbate endothelial damage and thrombotic risk [13,14,15]. Elevated triglycerides (TGs) have also been observed in critically ill patients, reflecting metabolic stress and altered lipid transport [7,16,17,18].

Despite these observations, the clinical relevance of lipid abnormalities in COVID-19 remains uncertain. Some researchers propose that lipid derangements may serve as severity markers, while others view them as non-specific consequences of inflammation [5,9,15,19]. Understanding these alterations is essential for their potential use in prognostic models and therapeutic targeting.

In this study, we primarily aimed to investigate the association between lipid profile components (LDL-C, HDL-C, and triglycerides) and COVID-19 severity. Our goal was to identify potential relationships between these lipid biomarkers and clinical outcomes, which may inform future research on prognostic models.

## 2. Materials and Methods

We conducted a retrospective study at AHEPA University General Hospital in Northern Greece, including adult patients hospitalized with confirmed SARS-CoV-2 infection between 1 October and 30 November 2021. The study protocol was approved by the Scientific Board of AHEPA (Approval No. 36002/29th/4 December 2024).

Eligible patients were ≥16 years old and had community-acquired COVID-19 confirmed by RT-PCR. We excluded those with in-hospital-acquired infections or missing laboratory data. A total of 208 patients were included in the final analysis.

Data were collected from electronic medical records and included demographic information, lipid profiles (total cholesterol, LDL-C, HDL-C, and triglycerides), inflammatory markers (CRP, ferritin, and IL-6), clinical outcomes (ICU admission and mortality), and the use of antilipidemic therapy prior to or during hospitalization. Severe COVID-19 was defined as the need for ICU admission and/or mechanical ventilation according to the WHO classification guidelines.

Comorbidities such as hypertension, diabetes mellitus, coronary artery disease, COPD, and chronic kidney disease were recorded. Obesity was defined according to the WHO criteria (BMI ≥ 30 kg/m^2^), and patients were classified as Class I (30.0–34.9), Class II (35.0–39.9), or Class III (≥40.0).

Statistical analysis was conducted using SPSS v.19. Continuous variables are presented as median and interquartile range (IQR). All continuous variables were tested for normality and found to be non-normally distributed; therefore, non-parametric statistical tests were applied. Group comparisons were performed using the Mann–Whitney U test, while associations between variables were assessed using Spearman’s rank correlation coefficient. A *p*-value < 0.05 was considered statistically significant.

## 3. Results

A total of 208 patients were included in the study. Baseline demographic, anthropometric, inflammatory, and lipid profile characteristics are presented in Table 1.

The median LDL-C level in the cohort was 77.0 mg/dL (IQR: 47.0–107.0). Antilipidemic treatment had been prescribed to 48.1% of discharged patients prior to hospitalization; however, it was not associated with significant differences in LDL-C levels or clinical outcomes.

A comparison between survivors and non-survivors revealed no statistically significant differences in LDL-C or HDL-C levels. Total cholesterol levels were also comparable. However, non-survivors had significantly higher triglyceride levels (median: 124.0 mg/dL vs. 108.0 mg/dL, *p* = 0.04) and CRP levels (median: 90 mg/L vs. 66 mg/L, *p* = 0.01) (Table 2).

### 3.1. Subgroup Analyses

#### 3.1.1. ICU Admission

Patients admitted to the ICU (*n* = 62) had lower median LDL-C levels [72.0 mg/dL (IQR: 44.0–100.0)] compared to non-ICU patients [78.0 mg/dL (IQR: 50.0–110.0)], although the difference was not statistically significant (*p* = 0.08).

#### 3.1.2. Obesity and Triglycerides

**Figure 1:**
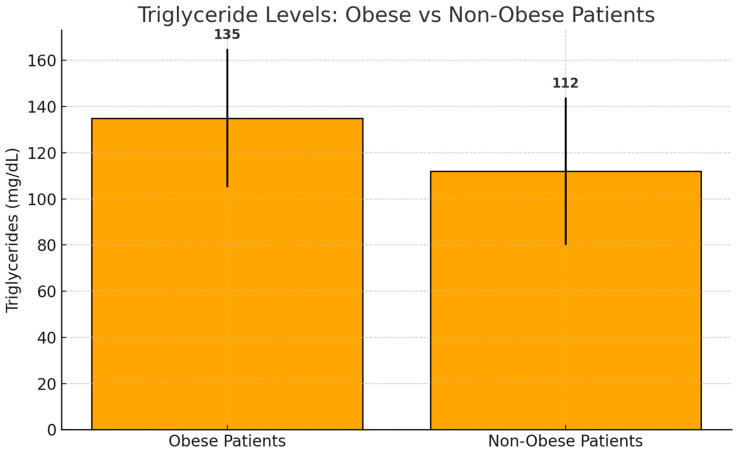
Median triglyceride levels in obese and non-obese patients. Bars represent median values with interquartile ranges (IQRs). Obese patients exhibited significantly higher triglyceride levels (median: 135.0 mg/dL, IQR: 98.0–170.0) compared to non-obese patients (median: 112.0 mg/dL, IQR: 80.0–138.0) (*p* = 0.03).

Obese patients (BMI ≥ 30 kg/m^2^) had significantly higher triglyceride levels [median: 135.0 mg/dL, IQR: 98.0–170.0] than non-obese patients [median: 112.0 mg/dL, IQR: 80.0–138.0] (*p* = 0.03) (Figure 1).

#### 3.1.3. Stratified Analysis of Triglycerides by Obesity Class

To further investigate the relationship between obesity and triglyceride levels, we conducted a subgroup analysis based on the WHO-defined obesity classes. Patients with Class I obesity (BMI 30.0–34.9 kg/m^2^) had a median triglyceride level of 129.0 mg/dL (IQR: 95.0–162.0), while those in Class II (BMI 35.0–39.9 kg/m^2^) had a median of 141.0 mg/dL (IQR: 110.0–178.0). Patients in Class III (BMI ≥ 40.0 kg/m^2^) exhibited the highest triglyceride levels, with a median of 152.0 mg/dL (IQR: 119.0–190.0). These findings suggest a progressive increase in triglyceride levels with rising obesity class.

Although the differences did not reach statistical significance, the observation that patients with a BMI ≥ 35 kg/m^2^ (Class II and III obesity) had even higher triglyceride levels than those with Class I obesity or a normal BMI supports the hypothesis that obesity amplifies dyslipidemic responses during acute COVID-19 infection. Further analysis with larger cohorts is warranted to validate these trends.

#### 3.1.4. CRP and HDL-C

In patients with elevated inflammation (CRP ≥ 10 mg/L), median HDL-C levels were significantly lower [33.0 mg/dL (IQR: 24.0–41.0)] compared to those with CRP < 10 mg/L [38.0 mg/dL (IQR: 30.0–47.0)] (*p* = 0.04).

#### 3.1.5. LDL-C Trends in Non-Survivors

Among non-survivors LDL-C levels declined during hospitalization, from a median of 76.0 mg/dL at admission to 66.0 mg/dL by day 7. However, this decrease did not reach statistical significance (*p* = 0.12).

#### 3.1.6. Longitudinal Follow-Up

Among discharged patients with available follow-up data LDL-C levels increased within four weeks post-recovery, with a median rise of +5.3 mg/dL compared to baseline. This change was statistically significant (*p* = 0.0019).

**Figure 2:**
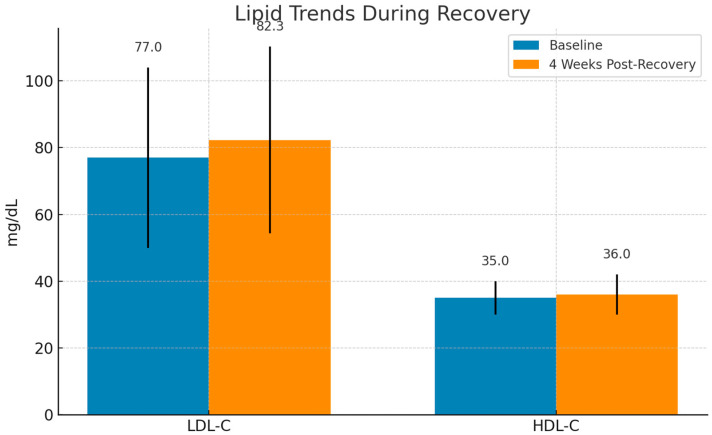
Evolution of lipid levels during recovery from COVID-19. LDL-C levels exhibited a significant increase (*p* = 0.0019), suggesting metabolic restoration, whereas HDL-C remained relatively suppressed with no significant change (*p* = 0.053). Median values are shown with interquartile ranges (IQRs).

In contrast, HDL-C levels remained relatively suppressed, with only minimal recovery. The difference compared to baseline did not reach statistical significance (*p* = 0.053), indicating persistent dysregulation in HDL-related anti-inflammatory function (Figure 2).

#### 3.1.7. Visual and Statistical Highlights

Significantly higher triglyceride levels were observed in obese compared to non-obese patients (*p* = 0.03), as previously shown.

Post-recovery trends revealed persistent HDL-C dysregulation despite LDL-C normalization, suggesting ongoing metabolic alterations.

**Figure 3:**
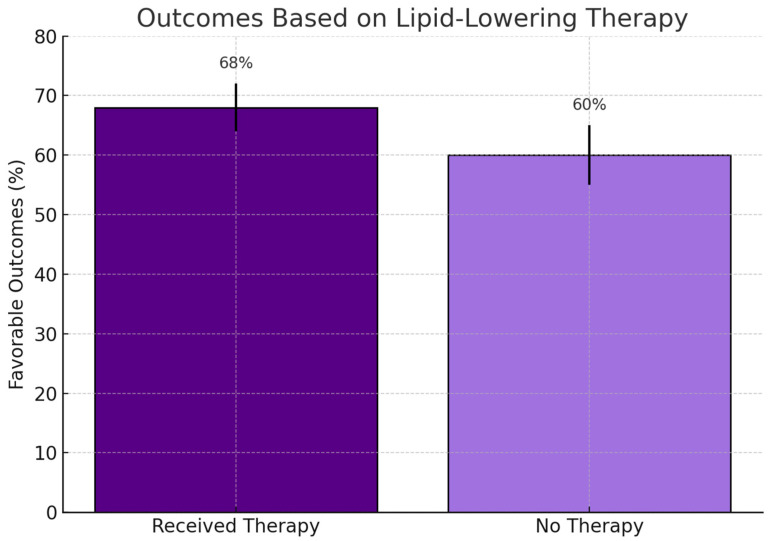
Clinical outcomes among patients who received lipid-lowering therapy versus those who did not. Bars represent favorable outcome percentages with standard error. Although a numerically higher rate was observed in the treated group (68% vs. 60%), the difference was not statistically significant (*p* = 0.30; χ^2^ test).

Figure 3 shows a trend toward improved clinical outcomes among patients receiving lipid-lowering therapy, although the difference did not reach statistical significance (*p* = 0.30).

## 4. Discussion

Although our study does not establish definitive prognostic cut-off values for lipid parameters, the observed associations suggest that lipid profile alterations may reflect the underlying inflammatory environment. Future prospective studies should incorporate stratified analysis and receiver operating characteristic (ROC) curves to assess the predictive value of lipid parameters more precisely.

Importantly, this study did not aim to define LDL-C cut-off values for prognostic purposes. Instead, we focused on describing associations with clinical severity. Future research, ideally using ROC analysis and larger, multi-center datasets, should explore the establishment of clinically relevant thresholds.

Our findings align with previous studies reporting lipid disturbances in patients with COVID-19. Reduced LDL-C and HDL-C levels, along with increased triglycerides, appear to reflect systemic inflammation rather than direct viral effects. Although these lipid changes were notable, they lacked prognostic significance in our cohort, highlighting the multifactorial nature of COVID-19 outcomes.

### 4.1. Comparison with Existing Literature

Several studies support the association between lipid profile alterations and COVID-19 severity. Tanaka et al. observed reduced LDL-C and HDL-C among ICU patients, correlating with inflammation markers such as CRP [6]. Similarly, Feingold and Li demonstrated that lower baseline HDL-C and LDL-C levels were associated with increased mortality [19,20]. Aung and Liang reported that genetic predispositions affecting LDL-C may influence susceptibility to severe disease [21,22]. The Apo COVID study further highlighted dynamic interactions between lipid profiles and inflammation in critically ill patients [6].

Other investigations, including those by Caterino et al. and Zhang Z. et al., employed lipidomic and immunologic profiling to reveal distinct alterations in lipid metabolism based on disease severity [14,16]. These findings collectively support the potential role of lipid markers not only as indicators of disease progression but also as active mediators in the pathophysiology of COVID-19.

### 4.2. Inflammation and Lipid Dynamics

The cytokine storm observed in COVID-19 drives extensive metabolic changes. Pro-inflammatory cytokines such as CRP, IL-6, and TNF-α have been inversely correlated with HDL-C and LDL-C levels [5,7,10,14], indicating that systemic inflammation directly disrupts lipid parameters. Several studies have documented that SARS-CoV-2 infection induces dyslipidemia, frequently characterized by decreased HDL-C and LDL-C concentrations and elevated triglycerides during the acute phase [14,23,24,25].

These alterations may stem from hepatic dysfunction, impaired lipoprotein synthesis, and increased lipid catabolism.

Interestingly, triglyceride levels may also decline in critically ill patients due to hepatic exhaustion, which adds complexity to the interpretation of lipid trends. Additionally, high membrane cholesterol levels have been shown to enhance viral entry by increasing ACE2 receptor density on host cells [26,27,28], further linking lipid dynamics to infection severity.

Reduced HDL-C and ApoA1 compromises anti-inflammatory and antioxidant defense mechanisms, potentially worsening endothelial injury, oxidative stress, and thrombotic complications in COVID-19. HDL-C and ApoA1 play critical roles in modulating vascular inflammation and oxidative stress through their capacity to neutralize oxidized lipids and inhibit adhesion molecule expression on endothelial cells [29,30,31]. Several studies have reported that lower levels of HDL-C and ApoA1 are associated with more severe COVID-19 outcomes, including thromboinflammation and endothelial dysfunction [6,8,32]. These immunometabolic disruptions may underlie poor clinical outcomes in vulnerable patient populations.

### 4.3. Clinical and Therapeutic Implications

Although LDL-C was not found to be an independent prognostic marker in our cohort, HDL-C and the TG/HDL-C ratio may serve as more robust indicators of severity, as emphasized by Zhang et al. [16]. Furthermore, the potential pleiotropic effects of statins—including endothelial protection and cytokine modulation—warrant further investigation. Current evidence suggests they may attenuate the inflammatory cascade and improve outcomes in hospitalized patients.

### 4.4. Integrated View and Public Health Relevance

Our results should be interpreted within the broader context of cardiometabolic health. Studies using large datasets, such as the UK Biobank, revealed that elevated LDL-C and TG levels increase the risk of infection, while higher HDL-C appears protective [25]. Ochoa-Ramírez et al. confirmed that lipid abnormalities were associated with worse ICU outcomes and prolonged hospitalization [33]. Hua et al. further emphasized the importance of addressing oxidative stress and metabolic dysregulation in COVID-19 management [34].

Taken together, these data reinforce the importance of integrating lipid profile assessment into personalized care strategies for COVID-19 patients, particularly those with underlying dyslipidemia or metabolic syndrome.

### 4.5. Strengths and Limitations

This study’s strengths include its real-world cohort and focus on both inflammatory and lipid biomarkers. However, there are limitations such as the relatively small patient population, the retrospective design of the study which predisposed it to biases, the absence of Kaplan–Meier survival analysis, and the lack of multivariable modeling (e.g., GLM, GEE). IL-6 and ferritin were not available for all patients and analyses of NLR and SII were not performed due to missing data. Moreover, there is a profound lack of longitudinal lipidomic profiling. These limitations are acknowledged, and the need for prospective, multi-center studies is emphasized.

### 4.6. Future Directions

Further research should explore the mechanistic pathways linking inflammation and lipid metabolism, integrating lipidomics and proteomics for comprehensive biomarker discovery. Longitudinal studies examining the impact of lipid-lowering therapies on inflammation and clinical recovery are also needed. This line of investigation may reveal novel therapeutic targets and strategies for improving outcomes in patients with COVID-19 and related viral illnesses.

## 5. Conclusions

This study adds to the growing body of literature exploring the role of lipid metabolism in COVID-19. Our analysis of LDL-C, HDL-C, and triglyceride levels revealed that although LDL-C was not independently associated with clinical outcomes, reductions in HDL-C and increases in triglycerides—particularly in obese and critically ill patients—suggest a potential interplay between the overall lipid profile and disease severity.

To our knowledge, this is the first study investigating these associations in a hospitalized cohort from Northern Greece, providing region-specific insights into the metabolic response to SARS-CoV-2 infection.

While our findings did not support the prognostic utility of LDL-C, they underscore the need to further explore lipid profiles as dynamic biomarkers of inflammation and recovery. Future prospective studies with broader datasets and advanced modeling approaches are essential to clarify the causal relationships between lipid dysregulation and COVID-19 outcomes, and to evaluate lipid-modulating therapies as potential adjuncts in clinical care.

## Figures and Tables

**Table 1:** Demographic and clinical data of the study population. Values are presented as medians (interquartile range). Antilipidemic therapy refers to treatment prior to hospitalization.

Variable	Median (IQR)	Min	Max
Age (years)	74.5 (62.0–86.0)	16.0	102.0
LDL (mg/dL)	77.0 (47.0–107.0)	11.0	200.0
HDL (mg/dL)	35.0 (27.0–43.0)	7.0	81.0
Triglycerides (mg/dL)	110.0 (77.0–143.5)	45.0	589.0
Total Cholesterol (mg/dL)	138.0 (111.0–165.0)	25.0	265.0
BMI (kg/m^2^)	28.6 (25.4–32.8)	18.9	43.1
CRP (mg/L)	70 (45–105)	3	215
Ferritin (ng/mL)	490 (290–710)	50	1380
IL-6 (pg/mL)	60 (35–92)	12	215
Antilipidemic Therapy (%)	48.1%	-	-

**Table 2:** Laboratory parameters by clinical outcome group. Values are presented as medians (interquartile range). Triglyceride and C-reactive protein (CRP) levels are significantly higher in non-survivors. IL-6 values were available for 68% of the study population. Differences are statistically significant at *p* < 0.05.

Variable	Median (IQR) Survivors	Median (IQR) Non-Survivors	Min	Max	*p*-Value
LDL (mg/dL)	78 (50–109)	72 (44–100)	12	200	0.24
HDL (mg/dL)	36 (28–44)	33 (25–41)	9	81	0.12
Triglycerides (mg/dL)	108 (75–136)	124 (89–168)	45	589	0.04 *
Total Cholesterol (mg/dL)	139 (112–166)	132 (109–160)	30	265	0.38
CRP (mg/L)	66 (40–101)	90 (63–130)	3	215	0.01 *
Ferritin (ng/mL)	460 (278–690)	560 (340–825)	50	1380	0.07
IL-6 (pg/mL) **	58 (34–90)	66 (38–101)	12	215	0.18

* Statistically significant at *p* < 0.05. ** IL-6 values were available for 68% of the study population.

## Data Availability

The data presented in this study are available on request from the corresponding author. The data are not publicly available due to privacy and ethical restrictions.

## Abbreviations

The following abbreviations are used in this manuscript:

ARDS	Acute Respiratory Distress Syndrome
BMI	Body Mass Index
COVID-19	Coronavirus Disease 2019
CRP	C-Reactive Protein
HDL-C	High-Density Lipoprotein Cholesterol
ICU	Intensive Care Unit
IL-6	Interleukin 6
IQR	Interquartile Range
LDL-C	Low-Density Lipoprotein Cholesterol
SD	Standard Deviation
SARS-CoV-2	Severe Acute Respiratory Syndrome Coronavirus 2
TC	Total Cholesterol
TGs	Triglycerides
